# Parental Feeding Practices, Child Eating Behaviours, and Their Association with Metabolic Control in Children with Type 1 Diabetes: A Systematic Review

**DOI:** 10.3390/pediatric18050129

**Published:** 2026-09-28

**Authors:** Raquel Oliveira, José Miguel Martinez-Sanz, João P. M. Lima, Marta García-Poblet, Isabel Sospedra

**Affiliations:** 1 Nutrition Department, Local Health of Leiria Region, Rua das Olhalvas, 2141-016 Leiria, Portugal; raquel.oliveira@ulsrl.min-saude.pt; 2 ciTechCare—Center for Innovative Care and Health Technology, Health Innovation Hub|Politécnico de Leiria, Campus 5, Rua das Olhalvas, 2414-016 Leiria, Portugal; 3 Higher School of Health, University of Algarve, Campus de Gambelas, 8005-139 Faro, Portugal; 4 Nursing Department, Faculty of Health Science, University of Alicante, 03690 Alicante, Spain; josemiguel.ms@ua.es (J.M.M.-S.); marta.poblet@ua.es (M.G.-P.); isospedra@ua.es (I.S.); 5 Research Group on Applied Dietetics, Nutrition and Body Composition (DANuC), University of Alicante, 03690 Alicante, Spain; 6 Polytechnic University of Coimbra, Rua da Misericórdia, Lagar dos Cortiços, S. Martinho do Bispo, 3045-093 Coimbra, Portugal; 7 CERNAS—Research Center for Natural Resources, Environment and Society, 3046-854 Coimbra, Portugal; 8 H&TRC—Health & Technology Research Center, Coimbra Health School, Polytechnic University of Coimbra, 3045-043 Coimbra, Portugal; 9 SUScita—Research Group for Sustainability, Cities and Urban Intelligence, Polytechnic University of Coimbra, 3030–199 Coimbra, Portugal

**Keywords:** type 1 diabetes, parental feeding practices, mealtime behaviour, family mealtime functioning, glycaemic control, paediatric diabetes, systematic review

## Abstract

Background/Objectives: Family-related factors are central to type 1 diabetes (T1D) management during childhood and adolescence. This systematic review aimed to synthesise the available evidence on the associations between parental feeding practices, child eating and mealtime behaviours, and glycaemic, nutritional and other diabetes-related outcomes in children and adolescents with T1D. Methods: This systematic review was conducted and reported in accordance with the PRISMA 2020 statement and prospectively registered in PROSPERO (CRD420261291514). PubMed/MEDLINE, Scopus, and Web of Science were searched without publication date restrictions. Observational studies including children and adolescents aged 0–18 years with T1D and examining parental feeding practices or child eating/mealtime behaviours in relation to diabetes-related outcomes were eligible. Family mealtime functioning was considered as a secondary construct when examined within otherwise eligible feeding- or mealtime-focused studies. Study quality and risk of bias were assessed using the Newcastle–Ottawa Scale. Owing to substantial heterogeneity in study populations, constructs, assessment instruments and outcomes, findings were synthesised narratively. Results: Eleven reports representing nine unique studies were included. Most studies were cross-sectional and involved predominantly preschool and younger school-aged children, with limited representation of adolescents. Associations between feeding and mealtime-related factors and diabetes-related outcomes varied across studies and constructs. Greater child mealtime problems and specific disruptive mealtime behaviours were associated with higher HbA1c or less favourable glucose-related measures in some analyses, while aspects of family mealtime functioning were associated with dietary adherence or glucose measurements outside the target range. Parental feeding practices, including restriction and pressure to eat, showed inconsistent or null associations with glycaemic outcomes. Associations were also reported with diet quality, carbohydrate intake and mealtime behaviours, although effect direction and magnitude varied, and null findings were common. Evidence from continuous glucose monitoring-derived outcomes was limited. Conclusions: Parental feeding practices and child mealtime behaviours may be associated with glycaemic, dietary and behavioural outcomes in paediatric T1D, while family mealtime functioning emerged as a relevant secondary contextual construct. However, the available evidence is heterogeneous and predominantly cross-sectional. These constructs should not be considered interchangeable dimensions of parental control, and current evidence does not establish directionality or causality, nor demonstrate that modifying feeding or mealtime practices improves glycaemic outcomes. Further longitudinal and intervention studies using clearly defined constructs, developmentally diverse samples, and objective glycaemic measures are needed.

## 1. Introduction

Type 1 diabetes mellitus (T1D) is a chronic metabolic disorder characterised by hyperglycemia resulting from autoimmune destruction of pancreatic β-cells, leading to absolute insulin deficiency [1].

T1D is one of the most frequent chronic conditions diagnosed during childhood and adolescence, and its incidence continues to rise globally [2]. Recent epidemiological data indicate that approximately 1.8 million individuals under the age of 20 years are currently living with T1D worldwide, with Europe reporting one of the highest regional prevalences. This growing burden places substantial demands not only on healthcare systems, but also on families, who play a central role in the daily management of the condition [3].

Effective paediatric diabetes management requires ongoing blood glucose monitoring, timely insulin administration, and consistent adherence to nutritional recommendations, particularly regarding carbohydrate counting and balanced meal composition. International paediatric diabetes guidelines emphasise that, especially in younger children, these complex and interdependent tasks depend almost entirely on parental supervision, planning and decision-making [4,5].

Consequently, parental practices surrounding food provision, mealtime routines and parent–child interaction patterns become integral components of diabetes care and may directly influence treatment adherence and metabolic outcomes [6].

Parental feeding practices, defined as specific strategies that parents use to influence their child’s eating, have been extensively described in the general paediatric population, where they are known to influence dietary quality, food preferences and self-regulation [7]. Similarly, child mealtime behaviours such as food refusal, tantrums and food selectivity have been identified as important determinants of dietary intake across childhood [8].

Evidence from other chronic paediatric conditions further highlights the importance of parental influence on behavioural and health-related outcomes [9,10]. Likewise, family-centred interventions underscore the relevance of involving caregivers in the management of complex treatment routines and health behaviours [11,12].

In the context of T1D, parental feeding practices and child eating behaviours acquire particular clinical relevance, as they interact directly with dietary adherence, insulin dosing, and glycaemic control. Evidence from paediatric T1D populations indicates that both maladaptive parental strategies and problematic child mealtime behaviours are common and clinically meaningful, constituting relevant barriers to optimal diabetes management [13,14]. In parallel, family mealtime functioning, including household routines, structure, behavioural control, and emotional climate during meals, has been identified as an important contextual factor in paediatric diabetes management [15]. Mealtimes represent a critical setting in which multiple components of diabetes self-care converge, including blood glucose monitoring, insulin administration, and carbohydrate intake [5,6].

Previous studies have explored relationships between parental feeding practices, child mealtime behaviours, family mealtime functioning and diabetes-related outcomes [7,8,15]. However, these constructs have often been examined separately, using heterogeneous measures and outcomes, which limits an integrated understanding of their relationships with diabetes management.

Previous reviews have addressed related but distinct aspects of parental and family involvement in paediatric T1D. Costa et al. [11] synthesised evidence on parental psychosocial factors in relation to glycaemic control, whereas Yamaguchi et al. [12] focused on parental involvement and glycaemic outcomes in adolescents. However, these reviews did not specifically integrate feeding-related parenting with child mealtime behaviours in relation to diabetes-related outcomes, while also considering family mealtime functioning as a relevant secondary contextual construct. A focused search for previous reviews did not identify a systematic review specifically addressing these feeding-related parenting and child mealtime constructs in paediatric T1D. Thus, the present review addresses a narrower and more specific gap within the existing literature.

Accordingly, this systematic review aimed to synthesise the available evidence on the associations between parental feeding practices, child eating and mealtime behaviours, and glycaemic, nutritional and other diabetes-related outcomes in children and adolescents with T1D.

## 2. Materials and Methods

### 2.1. Type of Study

This systematic review was conducted and reported in accordance with the Preferred Reporting Items for Systematic Reviews and Meta-Analyses (PRISMA) 2020 statement (Supplementary Table S1) [16]. It aimed to synthesise the available evidence on the association between parental feeding practices, child eating and mealtime behaviours, and metabolic control in children with T1D. The review protocol was developed a priori and prospectively, registered in the PROSPERO international prospective register of systematic reviews (https://www.crd.york.ac.uk/PROSPERO/view/CRD420261291514), on 23 January 2026, before the prespecified start date of the review (25 January 2026). The review followed rigorous methodological standards to ensure transparency, reproducibility, and scientific integrity.

### 2.2. Data Sources and Search Strategy

A comprehensive search strategy was developed to identify relevant studies across PubMed/MEDLINE, Scopus, and Web of Science. The original searches were conducted on 25 January 2026 and were updated on 14 September 2026 to identify any newly published eligible studies. To maximise the retrieval of studies related to the research aim, the search terms were defined to encompass the following key concepts: (1) parental feeding practices and control; (2) childhood and adolescence; and (3) type 1 diabetes mellitus (T1D). The strategy incorporated Medical Subject Headings (MeSH) descriptors, their corresponding entry terms (including historical terminology), and free-text keywords combined with the tags [tiab] or [Title/Abstract] to capture relevant occurrences within article titles and abstracts. The complete list of search terms and Boolean strategy is provided in Appendix B. The search was adapted for each database using the Polyglot Search Translator from the Systematic Review Accelerator tool (actual Tera Tools) [17]. No temporal restrictions were applied. To complement the electronic database searches, backward and forward citation searches (hand-screening reference lists of all included studies and relevant review articles, as well as tracking citing papers) were performed to ensure maximum retrieval of eligible studies. Backward citation searching was conducted by screening the reference lists of the included reports. Forward citation searching of the included reports was subsequently conducted using OpenAlex. Records identified through citation searching were deduplicated and screened against the predefined eligibility criteria. Citation searching was performed as a complementary identification method to maximise the retrieval of potentially relevant reports. This represented a departure from the registered protocol, which had specified that no additional identification methods would be used.

The search strategy was explicitly operationalized around parental feeding practices, feeding control, and eating behaviours as the primary constructs. Broader dimensions of family functioning, routines, and interactions were examined secondarily as they emerged within the context of these parent-led feeding behaviours, rather than as independent primary search blocks.

### 2.3. Eligibility Criteria

The inclusion and exclusion criteria were defined according to the Population, Exposure, Outcomes, and Study design (PEOS) framework and are summarised in Table 1. Eligible studies included children and adolescents aged 0–18 years with T1D and examined parental feeding practices or behaviours and/or child eating or mealtime behaviours in relation to diabetes related outcomes. Family mealtime functioning was considered as a secondary contextual construct when reported within studies otherwise meeting these feeding- or mealtime-focused eligibility criteria. Only published studies employing observational design, including single-centre and multicentre studies, were considered eligible. The exclusion criteria were as follows: (a) studies published in languages other than Spanish, Portuguese, French, or English; (b) narrative reviews, systematic reviews, or meta-analyses; and (c) studies including participants with food allergies or medical conditions requiring dietary restrictions that could independently influence eating or feeding behaviours. No temporal restrictions were applied, allowing the search to cover the broader historical development of paediatric diabetes research, including the period since the establishment of ISPAD [18].

### 2.4. Article Management Process

All references retrieved from the database searches were organised in separate folders according to the database of origin and then imported into the Systematic Review Accelerator platform’s deduplication tool [19]. A master folder was created to detect and remove duplicate entries based on the automated deduplication software. The final deduplicated dataset was exported in RIS format for use in the screening process by the research team.

### 2.5. Study Selection

All retrieved articles were screened in duplicate through Rayyan web application (MA, USA) [20]. The initial screening, based on titles and abstracts, was independently performed by two authors (RMO and JPML). In cases of disagreement, a third reviewer (JMMS or IS) was consulted to determine whether the articles should be included or excluded. Articles deemed potentially eligible were then subjected to a full-text review, conducted independently and in duplicate by the same two authors (RMO and JPML). For reports for which the full text was not directly available, additional retrieval attempts were made through the electronic databases and available bibliographic records. Despite repeated attempts, the full text of three reports could not be obtained, and these reports could therefore not undergo full-text eligibility assessment.

Studies that did not meet the predefined eligibility criteria were excluded, with reasons documented accordingly. Additional reviewers (JMMS, IS) were consulted as needed to resolve uncertainties regarding specific articles. The complete study selection process is summarised in Figure 1, presented as a PRISMA flow diagram.

### 2.6. Data Extraction

Data from the included studies were independently extracted in duplicate by two authors (RMO and JPML) using a piloted data extraction form specifically developed for this review. The extraction process was structured to capture both general study characteristics and detailed population-specific information, with population data further categorised into parent- and child-related variables.

A predefined framework guided the extraction of variables relevant to the review objectives. For each study, information was collected on the study objective, sample size, and country, as well as the validated instruments or scales used to assess parental feeding practices and behaviours. Parent-related data included descriptions of feeding behaviours, such as structured, coercive, permissive, or responsive approaches, as well as additional contextual or psychosocial factors, including stress levels or parental perceptions of child eating behaviours. Child-related data encompassed age, gender, duration since T1D diagnosis, and relevant eating behaviours, such as picky eating, food refusal, or mealtime tantrums. Glycaemic outcomes, particularly glycated haemoglobin (HbA1c) and, when available, continuous glucose monitoring-derived measures such as time in range (TIR), were extracted alongside nutritional, dietary, anthropometric, and other diabetes related outcomes. All extracted information was systematically organised into structured tables to enable comparison across studies. Data extraction was conducted manually and independently by both reviewers, with subsequent cross-checking to ensure accuracy and consistency.

### 2.7. Risk of Bias (Study Quality and Data Collection)

The methodological quality and risk of bias of the included studies were assessed by two independent researchers (MGP and JMMS) using the adapted Newcastle–Ottawa Scale (NOS) for cross-sectional observational studies [21]. A third reviewer was consulted to resolve discrepancies (ISL). The assessment used a nine-star scoring system covering three main domains: (i) selection, including sample representativeness, sample size, response rate, and ascertainment of exposure; (ii) comparability, based on consideration of potential confounding; and (iii) outcome assessment, including the method of outcome ascertainment and appropriateness of the statistical analysis. Reports were classified as having a low (7–9 stars), intermediate (4–6), or high (0–3) risk of bias.

Study quality and risk of bias judgments were explicitly integrated into the qualitative synthesis, assigning greater evidential weight to studies with adjusted multivariable models, representative sampling, and lower overall risk of bias.

Accordingly, aggregate NOS scores were used as descriptive summaries of methodological quality and were not considered a complete assessment of risk of bias for individual exposure–outcome associations.

### 2.8. Data Synthesis

Given the heterogeneity of the included reports in terms of study populations, feeding and mealtime constructs, measurement instruments and diabetes-related outcomes, findings were synthesised narratively. Results were organised according to glycaemic, nutritional/dietary, and behavioural or mealtime-related outcomes. Effect estimates, measures of precision, analytical approaches and null findings were extracted as reported in the original studies. No set of at least three independent studies was considered sufficiently comparable in terms of exposures, populations and outcome assessment to support a meaningful quantitative synthesis; therefore, meta-analysis was not performed.

## 3. Results

### 3.1. Study Selection

The database searches identified 2106 records (PubMed/MEDLINE, n = 611; Scopus, n = 853; Web of Science, n = 642). After removal of 451 duplicate records, 1655 records were screened by title and abstract, of which 1638 were excluded. Seventeen reports were sought for retrieval. Despite repeated attempts to obtain the full text through the electronic databases and available bibliographic records, three could not be retrieved, leaving 14 full-text reports assessed for eligibility. Three reports were excluded following full-text assessment: one because the exposure was outside the predefined scope of the review, one because it did not report an eligible exposure–outcome association, and one because the study design and outcomes did not address the review question. A total of 11 reports, representing nine unique studies, were included in the systematic review. The study selection process is presented in the PRISMA 2020 flow diagram (Figure 1). The updated searches conducted on 14 September 2026 retrieved 202 records from PubMed/MEDLINE, 42 from Scopus, and four from Web of Science. Screening of these records against the predefined eligibility criteria identified no additional eligible reports; therefore, the evidence base of the review remained unchanged.

Backward citation searching identified 363 references across the reference lists of the included reports, corresponding to 294 unique references after deduplication. Forward citation searching in OpenAlex identified 215 citing records, corresponding to 158 unique records after deduplication. Following screening against the predefined eligibility criteria, no additional eligible reports were identified through citation searching.

### 3.2. Characteristics of Included Studies

The 11 included reports, representing nine unique studies, examined parental feeding practices, child eating or mealtime behaviours, and/or family mealtime functioning in paediatric T1D populations. The characteristics of the included reports are summarised in Table 2, with participant and clinical characteristics presented separately in Table 3. The evidence was derived predominantly from cross-sectional observational studies. One report analysed baseline data from an intervention study, while another used a retrospective cross-sectional design.

The included reports were published between 2006 and 2025 and were conducted predominantly in North America and Europe [22,23,24,25,26,27,28,29,30,31,32]. Sample sizes ranged from 31 to 258 participants. Although the eligibility criteria encompassed children and adolescents aged 0–18 years, the included samples ranged from approximately 1 to 15 years of age and were predominantly composed of preschool and younger school-aged children, with limited representation of adolescents.

Among the included reports, five publications by Patton and colleagues originated from three distinct research cohorts. Patton et al. (2006) [22] and Patton et al. (2009) [23] analysed different constructs within the same cohort (Cohort A), whereas the two Patton et al. (2013) [25,26]. publications represented separate analyses of another cohort (Cohort C). The 2009 study of children receiving insulin pump therapy represented an independent cohort (Cohort B) [24]. These linked reports were therefore considered as originating from the same underlying study population where appropriate and were not interpreted as independent replications of evidence.

Constructs and Outcomes Assessed

The outcomes assessed across the included reports are summarised in Table 4. Considerable heterogeneity was observed in the constructs and outcomes evaluated. Parental and family-related measures included parental feeding practices and family mealtime functioning, while child-related measures encompassed eating and mealtime behaviours. Glycaemic outcomes were assessed primarily using HbA1c, with a smaller number of reports including continuous glucose monitoring (CGM), derived measures, or other glucose-related parameters. Nutritional and dietary outcomes, including dietary quality, carbohydrate intake or deviation, and dietary adherence, were considered separately from glycaemic outcomes. Other clinical and anthropometric outcomes were reported less consistently across studies.

Associations Between Feeding/Mealtime Factors and Outcomes

Associations between parental feeding practices, child mealtime behaviours, family mealtime functioning and diabetes-related outcomes varied across studies and outcome domains. Effect estimates, measures of precision, analytical approaches and null findings, as reported in the original studies, are summarised in Table 5. Given the heterogeneity of the constructs, measurement instruments and outcomes assessed, findings are presented narratively according to glycaemic, nutritional/dietary and behavioural or mealtime-related outcomes.

For glycaemic outcomes, several reports identified associations between mealtime-related behaviours or family functioning and HbA1c or glucose-related measures, although findings were not consistent across studies. Greater child mealtime problems were associated with higher HbA1c in some analyses, while specific disruptive mealtime behaviours and aspects of family functioning were associated with glucose values or the proportion of glucose measurements outside the target range. However, other parental feeding practices and mealtime behaviours showed no significant associations with HbA1c or glucose-related outcomes. Evidence from CGM-derived measures was limited, preventing robust conclusions regarding associations with glucose variability or time in range.

Associations were also reported for nutritional and dietary outcomes. Child mealtime problems were associated with lower diet quality in one report, while parent-reported mealtime problems were associated with greater deviation from prescribed carbohydrate intake. Specific parental feeding practices were associated with food-group intake in some studies; however, the direction and magnitude of these associations varied according to the feeding construct and dietary outcome examined. Several analyses did not identify significant associations, further indicating heterogeneity across studies.

Behavioural and mealtime-related findings similarly varied according to the construct assessed. Certain parental feeding practices were associated with child resistance, food aversion or overall mealtime disturbance, whereas other feeding practices showed no significant relationships with child mealtime behaviours. Family functioning was also associated with parent- and child-reported mealtime problems in some analyses. Overall, the findings suggest relationships between specific feeding and mealtime constructs rather than a uniform pattern across parental feeding practices.

Overall, the direction and strength of associations differed across constructs and outcomes, and null findings were common. The available evidence therefore did not demonstrate a consistent association between any single parental feeding practice or mealtime construct and glycaemic control across studies.

### 3.3. Study Quality Assessment

Study quality was evaluated using the Newcastle–Ottawa Scale (NOS) for cross-sectional studies (Table 6), where scores of 7–9, 4–6, and <4 indicate low, intermediate, and high risk of bias, respectively. Overall, all eleven included reports were rated as having an intermediate risk of bias (scores ranging from 5 to 6 out of 9). Risk-of-bias assessment was conducted at the report level, as publications derived from the same underlying study could differ in the analyses, outcomes, and reporting relevant to the domains assessed.

In terms of selection criteria, all reports recruited participants from specific clinical or research centres, potentially limiting the representativeness of their samples relative to the broader paediatric diabetes population. However, all reports employed validated instruments for data collection, ensuring acceptable measurement reliability.

Regarding comparability, seven of the eleven reports accounted for potential confounding variables in their analyses. For outcome assessment, all reports combined self-administered measures (e.g., questionnaires) with either objective data, such as medical records, glucose sensor data, or laboratory results, or independent evaluations conducted by trained professionals blinded to participant characteristics.

Eight reports combined self-report measures with objective record-based data, and three combined self-report measures with independent blinded assessments. Finally, all reports provided clear and sufficiently detailed descriptions of their statistical analyses, supporting the transparency and reproducibility of their findings.

## 4. Discussion

This systematic review synthesised evidence from 11 reports representing nine unique studies examining associations between parental feeding practices and child mealtime behaviours, and diabetes-related outcomes in children and adolescents with T1D, while also considering family mealtime functioning as a secondary contextual construct. Overall, the findings suggest that feeding and mealtime-related factors may be associated with glycaemic, nutritional, and behavioural outcomes; however, the direction and strength of these associations varied across constructs and studies [22,23,24,25,26,27,28,29,30,31,32].

Across the included reports, several parental feeding practices, child mealtime behaviours, and aspects of family mealtime functioning were associated with glycaemic or dietary outcomes. However, these associations were not uniform across reports and should be interpreted in light of differences in methodological robustness. Several findings were derived from small, selected clinical samples and unadjusted or minimally adjusted analyses and therefore constitute preliminary evidence. Child mealtime problems and specific disruptive mealtime behaviours were associated with higher HbA1c or glucose-related measures in some analyses, while aspects of family functioning were associated with carbohydrate intake or glucose measurements outside the target range. Other parental feeding practices, including restriction and pressure to eat, showed inconsistent or null associations with glycaemic outcomes [22,23,28,32].

The heterogeneity in assessment approaches is also relevant when interpreting these findings. CFQ-based studies conceptualised parental feeding practices through constructs such as restriction and pressure to eat, whereas BPFAS-based studies focused on parent- and child-reported mealtime problems. Observational approaches, including the MICS, captured additional dimensions of family mealtime functioning and parent–child interactions [23,24,25]. These constructs are related but not interchangeable, which may partly explain the variability in the direction and strength of the associations observed across studies [33,34,35,36,37].

The terminology used for these behavioural and conceptual constructs across the included studies is summarised in Appendix A.

From a mechanistic perspective, the observed associations may reflect several plausible pathways, although these mechanisms were not directly established by the included studies. First, coercive feeding practices may interfere with children’s ability to self-regulate food intake, potentially contributing to greater variability in carbohydrate consumption and postprandial glucose responses [22,26]. Second, conflictual mealtimes may contribute to stress and difficulties in coordinating food intake with insulin administration, through both behavioural and physiological pathways [36,38]. Third, persistent feeding conflict may be associated with difficulties in treatment adherence over time [36,38], consistent with broader models linking parental distress, monitoring, and glycaemic outcomes in youth with T1D [39].

The findings suggest that mealtime disruption may be associated with poorer dietary consistency and greater variability in carbohydrate intake, potentially complicating the coordination between food consumption and insulin administration. Behavioural difficulties during meals may therefore represent relevant challenges to daily diabetes management [28].

Associations with dietary quality were also observed. In Lobley et al., children’s unwillingness to try new foods and longer meal duration were associated with lower vegetable intake. These findings highlight a potential relationship between feeding and mealtime behaviours and dietary quality. Less adaptive feeding strategies were also associated with less favourable dietary patterns, including lower vegetable intake and greater consumption of discretionary foods [30].

However, these findings also indicate a more complex and nuanced pattern of associations. Lobley et al. distinguished between overt and covert restriction. In unadjusted analyses, reward-based feeding was associated with less favourable dietary indicators, whereas covert restriction was more frequently reported among children achieving the recommended HbA1c target [30]. These findings should be interpreted cautiously, as the analyses did not account for potential confounding and therefore do not establish an independent association between these feeding practices and glycaemic or dietary outcomes [30]. This finding challenges a binary interpretation of parental “control” as uniformly detrimental and highlights the importance of distinguishing between different feeding constructs. Structured parental management implemented within a responsive and developmentally appropriate context may differ substantially from coercive or conflict-generating feeding practices [40].

Child mealtime behaviours were associated with glycaemic and dietary outcomes in several reports, although findings were not consistent across behaviours or studies. Food refusal, distractibility, prolonged meal duration and other mealtime difficulties were associated with less favourable glucose-related or dietary outcomes in some analyses [22,23,27]. However, the available evidence does not establish their independent contribution, and these associations should be interpreted within a potentially bidirectional framework. Child mealtime difficulties or suboptimal glycaemic outcomes may elicit more controlling or restrictive parental responses, while parental feeding behaviours may, in turn, shape child eating and mealtime interactions. Diabetes-related challenges, including concerns about glycaemic excursions, variable appetite and the burden of coordinating food intake with insulin therapy, may further influence parental responses at mealtimes. Given the predominance of cross-sectional evidence, the temporal sequence of these relationships cannot be established, and reverse causality remains a plausible explanation for some of the observed associations [36,41].

At the family level, mealtime emotional climate and structural organisation may represent relevant contextual factors in diabetes management [42]. Some reports found associations between aspects of family functioning, such as lower conflict or greater organisation, and more favourable glycaemic or dietary outcomes [23,25], whereas other family or feeding constructs showed weaker or null associations [23,29]. These findings are consistent with family-systems perspectives and contemporary paediatric diabetes frameworks, which recognise diabetes management as embedded within relational and organisational contexts rather than as an individual behaviour alone [4,5,33,43,44].

Parental psychosocial and motivational factors may further contextualise feeding and mealtime practices. In López-Morago et al., unadjusted correlation analyses identified weak associations between selected parental food-choice motives and metabolic or anthropometric outcomes; for example, greater importance placed on food familiarity was weakly associated with higher HbA1c [31]. Given the unadjusted nature and small magnitude of these associations, these findings should be regarded as preliminary.” [31]. More recent studies also included culturally diverse populations, although the available evidence did not demonstrate a consistent association between cultural background and glycaemic outcomes [30]. These findings suggest that feeding and mealtime practices should be considered within the broader family, cultural and socioeconomic context, while avoiding assumptions based on cultural background alone [45,46] Psychosocial, medical and demographic factors may also be associated with parental mealtime behaviours in families of young children with T1D [47].

The present review extends the previous literature addressing parental psychosocial factors [11], parental involvement in adolescents [12], and broader behavioural research in young children with T1D [41] by focusing specifically on feeding-related parenting and child mealtime behaviours, while considering family mealtime functioning as a secondary contextual construct within the same systematic evidence synthesis. By integrating these related but distinct constructs, the present review highlights their heterogeneity and indicates that they should not be interpreted as a single dimension of parental control. Importantly, the available evidence does not allow the independent contribution of specific feeding or mealtime behaviours to glycaemic outcomes to be established, and the predominance of cross-sectional designs prevents conclusions regarding directionality or causality.

Taken together, the findings suggest that parental feeding practices, child mealtime behaviours and family mealtime functioning are interrelated factors that may be associated with glycaemic, dietary and behavioural outcomes in paediatric T1D. Importantly, the evidence also highlights the need to distinguish supportive structure and responsive guidance from coercive or conflict-generating feeding practices, rather than considering parental control as a single construct. This distinction is consistent with contemporary recommendations supporting family-centred approaches to paediatric diabetes management [4,5,33,48,49].

From a clinical perspective, the current evidence is more informative for identifying mealtime difficulties and generating hypotheses for family-centred behavioural interventions than for supporting specific changes in feeding practices. Assessment of problematic mealtime behaviours and family feeding dynamics may help identify families who could benefit from additional multidisciplinary support. However, given the uncertainty regarding directionality and the absence of evidence that modifying specific feeding practices improves glycaemic outcomes, prescriptive recommendations cannot currently be justified [50].

These findings should be interpreted considering several methodological limitations. Comparatively greater evidential weight was given to findings derived from analyses that accounted for relevant covariates, although these results remain observational and, predominantly, cross-sectional. Accordingly, even adjusted associations cannot establish directionality or causality. The predominance of cross-sectional designs precludes causal inference and limits the ability to establish temporal relationships between feeding or mealtime factors and diabetes-related outcomes [22,23,24,25,26,27,28,29,30,31,32]. Sample sizes were generally small, and several reports were derived from overlapping participant cohorts, reducing the number of independent evidence units and limiting the strength of apparent replication across studies. Measurement heterogeneity, including the use of different instruments to assess related but non-equivalent constructs, further limits direct comparison across studies. In addition, reliance on parent-reported measures in several studies may have introduced reporting bias. Given these limitations, together with the variability and null findings observed across outcomes, the direction and magnitude of the reported associations should be interpreted cautiously. Importantly, the included reports should not be regarded as contributing equivalent evidential weight to the synthesis. Differences in sample size and representativeness, control of confounding, and analytical approach affect the confidence that can be placed in individual associations.

A further consideration relates to developmental stage. Although the eligibility criteria encompassed children and adolescents aged 0–18 years, the available evidence was predominantly derived from preschool and younger school-aged children, with limited representation of adolescents. Feeding and mealtime interactions are likely to differ across developmental stages as children acquire greater autonomy in food choice and diabetes self-management [50].

However, the limited adolescent evidence and the absence of consistent age-stratified analyses prevent conclusions regarding whether the observed associations differ across childhood and adolescence. The findings of this review should therefore be interpreted primarily in relation to younger paediatric populations, and extrapolation to adolescents should be made cautiously.

Future research should prioritise longitudinal designs to clarify the temporal and potentially bidirectional relationships between parental feeding practices, child mealtime behaviours, family mealtime functioning and diabetes-related outcomes. Studies should use clearly defined and conceptually distinct feeding and mealtime constructs, adequately powered and independent samples, and objective glycaemic measures, including CGM-derived metrics such as time in range and glycaemic variability. Age-stratified analyses are also needed to determine whether these associations differ across developmental stages, particularly during adolescence, which remains underrepresented in the current evidence. Future studies should further examine the relative and independent contributions of parental, child and family-level factors while accounting for relevant clinical and psychosocial confounders. Ultimately, intervention studies will be required to determine whether modifying feeding or mealtime-related factors can improve glycaemic, dietary or behavioural outcomes in children and adolescents with T1D.

## 5. Conclusions

This systematic review suggests that parental feeding practices, child eating and mealtime behaviours may be associated with glycaemic, dietary and behavioural outcomes in children and adolescents with T1D. Family mealtime functioning also emerged as a relevant secondary contextual construct within the included feeding- and mealtime-focused studies.

Across 11 reports representing nine unique studies, these factors were variably associated with glycaemic, dietary and behavioural outcomes, with several analyses also reporting null findings. Importantly, the findings highlight the heterogeneity of feeding and mealtime constructs and indicate that supportive structure, coercive feeding practices, child mealtime behaviours and broader family functioning should not be considered interchangeable dimensions of parental control.

The available evidence is predominantly cross-sectional and derived from relatively small samples, with substantial heterogeneity in constructs, measurement approaches and outcomes. Moreover, the evidence largely reflects preschool and younger school-aged, limiting generalisability to adolescents. Consequently, the current evidence supports associations but does not establish their directionality or causality, nor does it demonstrate that modifying parental feeding or mealtime practices improves HbA1c or other glycaemic outcomes.

Feeding and mealtime difficulties may nevertheless represent clinically relevant contextual factors that could be considered as part of a broader nutritional and behavioural assessment of families living with paediatric T1D. Longitudinal and intervention studies using clearly defined constructs, independent and developmentally diverse samples, and objective glycaemic measures are needed to clarify these relationships and determine whether feeding- or mealtime-focused interventions can improve glycaemic, dietary or behavioural outcomes.

## Figures and Tables

**Figure 1:**
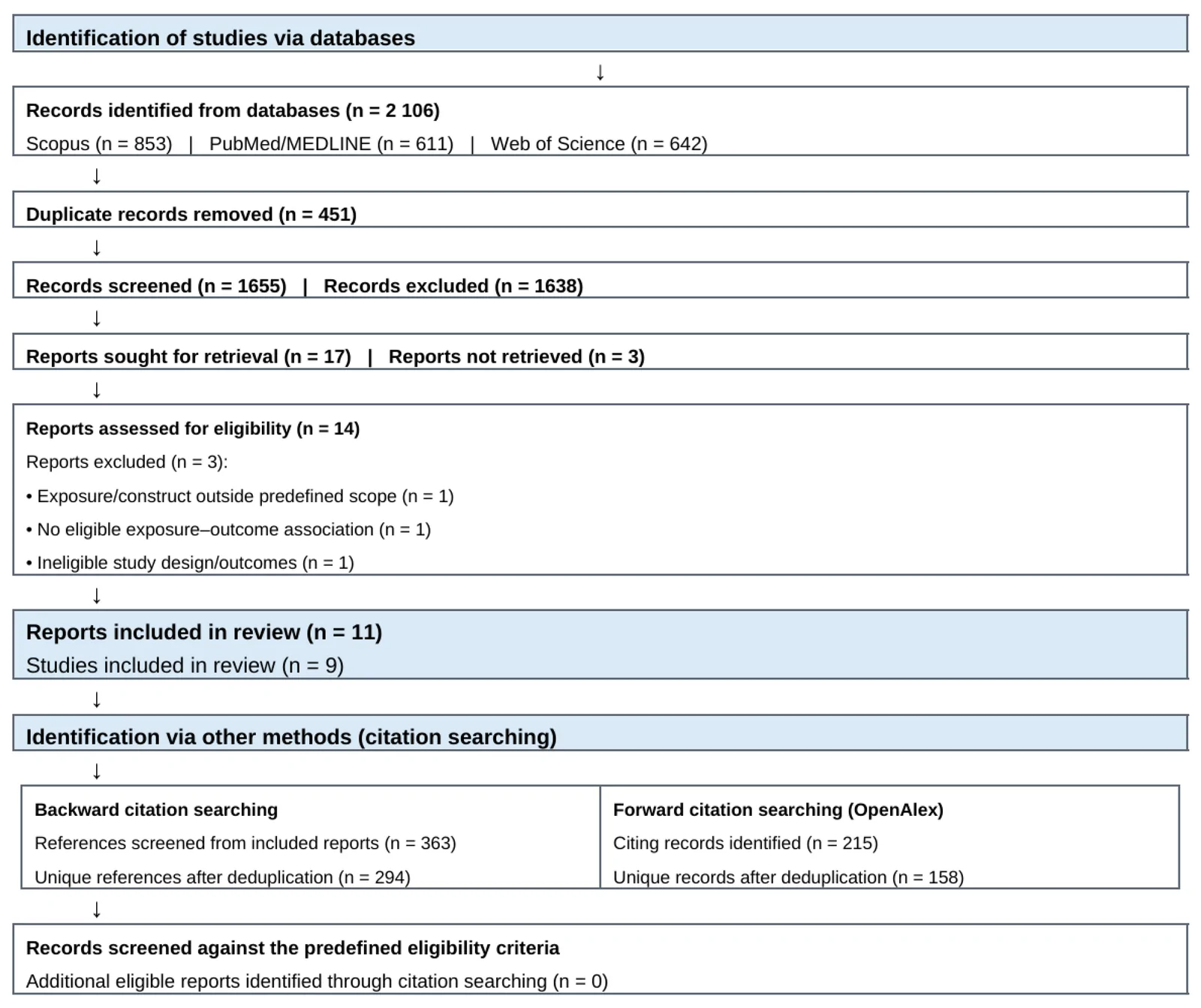
PRISMA 2020 flow diagram of study selection.

**Table 1:** The inclusion criteria applied in the study followed the population, exposure, outcomes and study design (PEOS) strategy.

Population	Exposure	Outcomes	Study Design
Children and adolescents aged 0–18 years with T1D	Primary constructs: Parental feeding practices and behaviours; child eating or mealtime behaviours. Secondary contextual construct: Family mealtime functioning, when assessed within otherwise eligible feeding- or mealtime-focused studies.	Primary outcome: Glycaemic/metabolic control parameters, particularly HbA1c; additional glycaemic outcomes, including time in range, were considered when available. Secondary outcomes: Dietary/nutritional and behavioural/mealtime related outcomes when available.	Observational studies (cross-sectional) and correlational studies

**Table 2:** Study characteristics.

Study	Recruitment/Period Setting	Cohort/Sample Overlap	Study Design	Study Objective	Sample Size	Inclusion/Exclusion Criteria	Assessment Instruments Used
Patton et al., 2006 [22]	~18 months (dates NR); Pediatric Diabetes Center, Cincinnati Children’s Hospital Medical Center, Cincinnati, OH, USA	Cohort A	Observational, Correlational Study	To examine the relationship between parent–child mealtime interactions and dietary adherence and glycaemic control in young children with T1D	35	Children aged 2–8 years, diagnosed with T1D for at least 1 year, no other conditions affecting growth, English-speaking families.	Dyadic Interaction Nomenclature for Eating (DINE), Behavioral Pediatrics Feeding Assessment Scale (BPFAS)
Patton et al., 2009 [23]	Dates NR; Pediatric Diabetes Center, tertiary hospital, Midwestern USA	Cohort A	Observational, Correlational Study	To examine the associations between family functioning at mealtimes and dietary adherence and glycaemic control in young children with T1D.	35	Diagnosed with T1D for at least 1 year, no other growth-affecting conditions; English-speaking households.	Videotaped meals, McMaster Interaction Coding System (MICS)
Patton et al., 2009 (Pump Therapy) [24]	Dates: NR; two Pediatric Diabetes Centers, Midwestern USA	Cohort B	Cross-sectional Observational Study	To assess parent-reported mealtime behaviours in young children with T1D on insulin pump therapy and examine correlations with glycaemic control.	31	T1D diagnosis > 1 year; using insulin pump; English-speaking families.	Child Feeding Questionnaire (CFQ), BPFAS
Patton et al., 2013 [25]	Dates: NR; two children’s hospitals, Midwestern USA	Cohort C	Cross-sectional Observational Study	To examine mealtime behaviours in families of young children with T1D on intensive insulin therapy and assess associations with glycaemic control.	39	T1D diagnosis > 1 year; intensive insulin therapy; English-speaking families.	DINE
Patton et al., 2013 (J Acad Nutr Diet) [26]	Dates: NR; Cincinnati Children’s Hospital and C.S. Mott Children’s Hospital, USA	Cohort C	Cross-sectional Observational Study	To examine dietary adherence, mealtime behaviours, and glycaemic control in young children with T1D on intensive insulin therapy.	39	T1D diagnosis > 1 year; intensive insulin therapy; English-speaking families	BPFAS, Dietary Deviation Scale, Healthy Eating Index 2005
Monaghan et al., 2015 [27]	Dates: NR; three tertiary diabetes care sites, USA	-	Cross-sectional Observational Study	To evaluate associations among diabetes-specific parent functioning, parent and child mealtime behaviours, and glycaemic control in young children with T1D.	134	Children with T1D and their parents; no detailed criteria specified.	BPFAS, hypoglycaemic fear, diabetes self-efficacy, diabetes-related Quality of Life scales
Mackey et al., 2020 [28]	Dates: NR; diabetes clinics in a metropolitan area, USA	-	Cross-sectional (baseline data from intervention trial)	To assess parent feeding behaviour, child eating behaviour, and nutrition intake in young children with T1D compared to guidelines.	46	Inclusion: children aged 2–5; T1D diagnosis > 1 year; parent ≥ 21 years, English-speaking. Exclusion: developmental disability or co-occurring serious illness.	BPFAS
Kelleher et al., 2023 [29]	Dates: NR; paediatric diabetes clinic, academic medical centre, Appalachian region, USA	-	Cross-sectional Observational Study	To examine associations among permissive parenting, mealtime behaviours, nutrition, and HbA1c in children with T1D.	63	Children with T1D and their caregivers; no detailed criteria provided.	Block Kids Food Screener (BKFS), About Your Child’s Eating Revised (AYCE-R), Parenting Styles and Dimensions Questionnaire–Short Version (PSDQ)
López-Morago et al., 2023 [30]	Feb–May 2022; Paediatric Endocrinology Unit, Puerta del Mar University Hospital, Cádiz, Spain	-	Cross-sectional Observational Study	To evaluate associations between parental food choice motivations and the nutritional status and glycaemic control of children with T1D.	85	T1D diagnosis > 1 year; children with complete sociodemographic and clinical data.	Food Choice Questionnaire (FCQ) (Spanish)
Catamo et al., 2024 [31]	Dates: NR; multicentre paediatric diabetes centres, Italy and Slovenia	-	Cross-sectional, Observational Study	To evaluate food behaviour and metabolic characteristics in children and adolescents with T1D and their impact on glycaemic control.	258	Children/adolescents aged 6–15 years, T1D diagnosis > 1 year; attending paediatric endocrinology units.	Child Food Questionnaire (CFQ) Italian Children Food Neophobia Scale
Lobley et al., 2025 [32]	Aug 2017–Oct 2019; three paediatric diabetes centres, Sydney, NSW, Australia	-	Retrospective, cross-sectional, multi-centre study	To examine responsive feeding practices in culturally diverse children aged 1–5 years with T1D and explore associations with glycaemic control, weight status and diet quality.	76	Inclusion: Children aged 1–5 years; T1D duration >3 months; Exclusion: Medical/developmental disorders affecting growth or feeding	Family Questionnaire (FQ) Feeding Practices and Structure Questionnaire-28 (FPSQ-28) Young Children’s Food and Drink Study Questionnaire

**Table 3:** Participant and clinical characteristics.

Study (Author, Year, Cohort)	Mean Age (or Range)	Gender (%)	T1D Duration	Treatment/Technology	Parents/Caregiver Characteristics
Patton et al., 2006 (cohort A) [22]	5.6 ± 1.6 years	40% male, 60% female	43.5 ± 22.9 months	Conventional insulin therapy: 27/35 (77%); intensive therapy: 8/35 (23%; MDI n = 1, pump n = 7)	Parents, further demographic characteristics: NR
Patton et al., 2009 (cohort A) [23]	5.6 ± 1.6 years	40% male, 60% female	>1 year	Conventional insulin therapy: 27/35 (77%); intensive therapy: 8/35 (23%; MDI n = 1, pump n = 7)	Parents, further demographic characteristics: NR
Patton et al., 2009 (Pump Therapy) (cohort B) [24]	5.0 ± 1.3 years	55% male	>1 year	Insulin pump therapy: 100%	Primary caregivers: 96% mothers
Patton et al., 2013 (cohort C) [25]	5.1 ± 1.1 years	51% male	>1 year	Intensive insulin therapy: 100%; pump 62%, remainder MDI; blinded CGM used for study assessment	One parent per child; further demographic characteristics NR
Patton et al., 2013 (J Acad Nutr Diet) (cohort C) [26]	5.1 ± 1.1 years	51% male	>1 year	Intensive insulin therapy: 36%; pump: 64%	Parents, further demographic characteristics: NR
Monaghan et al., 2015 [27]	5.32± 1.34 years	49% female	1.99 ± 1.24 years	72% basal–bolus or insulin pump; remaining participants on non-intensive regimens	Primary caregivers: 90% female
Mackey et al., 2020 [28]	4.72 ± 0.86 years	63% male	>1 year	Pump 27.3%; CGM 67.4%; 78% used carbohydrate counting for insulin dosing	Parents: 91.3% female; mean age 36.5 ± 6.2 y
Kelleher et al., 2023 [29]	10.3 ± 1.9 years	61.9% female	>6 months	Pump 61.9%; MDI 38.1%; CGM 68.3%	Caregivers: 98.4% female; 93.7% biological parents; mean age 38.3 ± 6.3 years
López-Morago et al., 2023 [30]	12.35 ± 3.07 years	56.5% female	6.5 ± 2.1 years	NR	Main caregivers: 95.3% female; 94.1% mothers; mean age 43.9 ± 7.2 y
Catamo et al., 2024 [31]	12.0 ± 2.6 years	52% male	4.9 ± 3.2 years	NR	Parents completed the CFQ; parent/caregiver age/sex: NR
Lobley et al., 2025 [32]	4.1 ± 1.2 years	54% female	1.4 ± 1.1 years	MDI 57.9%; insulin pump 42.1%; CGM 51.3%	80.3% mothers, 17.1% fathers, 2.6% other; predominantly aged 35–44 y (57.9%)

**Table 4:** Assessed outcomes.

Study (Author, Year, Cohort)	Parental Feeding/Family Construct	Child/Mealtime Construct	Glycaemic Outcomes	Nutritional/Dietary Outcomes	Other Outcomes
Patton et al., 2006 (A) [22]	Parent mealtime behaviours/ineffective or coercive feeding behaviours	Child disruptive and eating behaviours during meals	Mean daily blood glucose	Dietary adherence: carbohydrate additions/deletions; calorie intake	—
Patton et al., 2009–Family Functioning (A) [23]	Family mealtime functioning: task accomplishment, communication, affect management, interpersonal involvement, behavioural control, roles, general functioning	—	Daily blood glucose; % readings below/above target range	Dietary adherence: carbohydrate additions/deletions	—
Patton et al., 2009–Pump Therapy (B) [24]	Parental restriction; pressure to eat; parent mealtime behaviours/problems	Child mealtime behaviour/problems	HbA1c	—	—
Patton et al., 2013–Eat Behav (C) [25]	Ineffective parent mealtime behaviours	Child disruptive/eating behaviours	Mean CGM glucose; postprandial glucose	Carbohydrate intake	—
Patton et al., 2013–JAND (C) [26]	Parent-reported mealtime behaviours/problems	Child mealtime behaviours/problems	Mean daily CGM glucose	Dietary adherence (carbohydrate and calorie deviation); diet quality (HEI-2005)	BMI percentile
Monaghan et al., 2015 [27]	Diabetes-specific parent functioning: hypoglycaemia fear, diabetes self-efficacy, diabetes-related quality of life; parent mealtime behaviours	Child mealtime behaviours/problems	HbA1c; % blood glucose values outside target (<70 or >200 mg/dL)	—	—
Mackey et al., 2020 [28]	Parent feeding behaviours/feelings about mealtimes	Child eating/mealtime behaviours	HbA1c	Breakfast macronutrient composition (% carbohydrate, protein, fat); glycaemic index	BMI percentile
Kelleher et al., 2023 [29]	Permissive parenting; parent aversion to mealtime	Child resistance to eating; overall mealtime disturbance	HbA1c	Dietary intake/diet quality, including vegetable intake	—
López-Morago et al., 2023 [30]	Parental food-choice motivations	—	HbA1c; ambulatory glucose profile metrics including TIR	—	Anthropometry/body composition; lipid profile and other biochemical parameters
Catamo et al., 2024 [31]	Parental feeding practices: perceived responsibility, concern about weight, restriction, pressure to eat, monitoring	Food neophobia; food liking/preferences	HbA1c	Food liking/preferences by food group	BMI SDS; fasting lipid profile
Lobley et al., 2025 [32]	Responsive/non-responsive parental feeding practices	Child feeding behaviours; mealtime structure/environment	HbA1c	Dietary intake; diet quality/adherence to Australian dietary guidelines; snacking	BMI/BMI z-score; weight status

**Table 5:** Associations and effect estimates.

Study (Author, Year, Cohort)	Exposure/Outcome	Analysis/Adjustment/Analytic Sample	Effect Estimate/Precision	Direction and Interpretation	Null Findings
Patton et al., 2006 (A) [22]	Child noncompliance and away-from-table behaviour→mean glucose; parent coaxing/interrupted commands→carbohydrate deletions and mean glucose; physical prompts→mean glucose	Pearson and partial correlations; selected analyses controlled for child height, weight and/or time since diagnosis, as applicable; α = 0.01. Total sample n = 35; analytic n for individual associations: NR. Missing-data handling: NR.	Child noncompliance: r = 0.55; away from table: r = 0.61; coaxing→CHO deletions: r = 0.80; interrupted commands→CHO deletions: r = 0.64; coaxing→glucose: r = 0.58; interrupted commands→glucose: r = 0.66; physical prompts→glucose: r = 0.45; all *p* ≤ 0.01	Problematic child and ineffective/coercive parent mealtime behaviours were associated with greater dietary deviation and/or higher mean glucose.	Food refusal was not significantly associated with dietary adherence or mean glucose; child mealtime behaviours were not significantly associated with mean calorie intake.
Patton et al., 2009–Family Functioning (A) [23]	MICS Task Accomplishment/Behavioural Control→carbohydrate deletions; Affect Management→BG below target	Pearson correlations; unadjusted. Total sample n = 35; dietary-adherence analyses n = 27 (children receiving conventional insulin therapy); analytic n for individual glycaemic associations: NR. Missing-data handling NR.	Task Accomplishment: r = −0.43; Behavioural Control: r = −0.54; Affect Management: r = −0.33, *p* = 0.05	Better family mealtime functioning was associated with fewer carbohydrate deletions; better affect management was associated with fewer BG readings below target.	No MICS dimensions were significantly associated with carbohydrate additions; most dimensions were not significantly associated with glycaemic outcomes.
Patton et al., 2009–Pump Therapy (B) [24]	Child mealtime problems→HbA1c; parental restriction→HbA1c	Pearson correlations; unadjusted; α = 0.01. Total sample n = 31; analytic n for individual associations: NR. Missing-data handling NR.	Child Problem Number: r = 0.42, *p* = 0.01; Child Problem Frequency: r = 0.48, *p* = 0.007	More frequent/problematic child mealtime behaviours were associated with higher HbA1c.	Parental restriction was not significantly associated with HbA1c.
Patton et al., 2013–Eat Behav (C) [25]	Child play/away from table behaviour and ineffective parent mealtime behaviours→mean CGM/postprandial glucose	Pearson correlations; within-subject correlations for postprandial analyses; α = 0.01. Total sample n = 39; analytic n for individual associations and number of observations contributing to postprandial analyses: NR. Missing-data handling: NR.	Child play→mean glucose: r = 0.50, *p* < 0.01; away from table→mean glucose: r = 0.45, *p* < 0.01	Disruptive child mealtime behaviours were associated with higher mean CGM glucose.	Ineffective parent behaviours were not significantly associated with mean glucose; neither disruptive child nor ineffective parent behaviours were significantly associated with postprandial glucose.
Patton et al., 2013–JAND (C) [26]	Child mealtime problems→diet quality; parent mealtime problems→carbohydrate deviation; diet quality→mean glucose	Pearson correlations; unadjusted; α = 0.01. Total sample n = 39; analytic n for individual associations: NR. Diet records were paired with CGM data, and only complete matched days were retained for final analyses; CGM data loss was mainly due to sensor or user error.	Child Problem→HEI-2005: r = −0.40, *p* = 0.01; Parent Problem→CHO deviation: r = 0.43, *p* = 0.01; HEI-2005→mean glucose: r = −0.34, *p* < 0.05	More child mealtime problems were associated with poorer diet quality; more parent-reported problems were associated with greater carbohydrate deviation.	HEI-2005–glucose association did not meet the prespecified α = 0.01 threshold; several other BPFAS–diet associations were non-significant.
Monaghan et al., 2015 [27]	Child/parent mealtime problems and diabetes-specific parent functioning→BG out of range/HbA1c	Structural equation modelling; adjusted for child age, race, parent marital status, and insulin regimen; robust maximum likelihood estimation. Total sample n = 134; FIML used to handle missing data under a missing-at-random assumption.	Child problems→BG out of range→HbA1c: indirect *p* = 0.049; parent problems→BG out of range: β = −0.24, *p* = 0.05; parent functioning→child/parent problems: β = 0.33/0.53, both *p* < 0.001	Child mealtime problems were indirectly associated with higher HbA1c through out-of-range BG; poorer diabetes-specific parent functioning was associated with more child and parent mealtime problems.	Parent mealtime problems→HbA1c indirect association was non-significant (*p* = 0.06); parent functioning had no significant direct association with glycaemic-control indices.
Mackey et al., 2020 [28]	Breakfast macronutrient composition→HbA1c target status; insulin regimen × HbA1c status→breakfast protein intake	Independent-samples *t*-test and factorial ANOVA; unadjusted. Total sample n = 46; analytic n = 44 for the HbA1c-target group comparison (*t*-test); analytic n for factorial ANOVA: NR. Missing-data handling: NR.	CHO: 43.4% vs. 50.6%, t(42) = −1.91, *p* = 0.06; regimen × HbA1c status for protein: F(1,40) = 6.37, *p* = 0.02	The relationship between breakfast protein intake and HbA1c target status differed according to insulin regimen.	Carbohydrate difference by HbA1c status was non-significant (*p* = 0.06); no significant differences for protein, fat or glycaemic index, and no regimen × HbA1c interactions for carbohydrate, fat or glycaemic index.
Kelleher et al., 2023 [29]	Permissive parenting→vegetable intake and mealtime problems; permissive parenting→child resistance→HbA1c	Pearson correlations; PROCESS Model 4 mediation analyses adjusted for gender (vegetable-intake model) or CGM use and family income (HbA1c model). Analytic n = 63; no missing data for primary study variables.	Vegetable intake: r = −0.27, *p* = 0.03; child resistance: r = 0.48; parent aversion: r = 0.51; overall disturbance: r = 0.48, all *p* < 0.01; indirect effect on HbA1c non-significant	Greater permissive parenting was associated with lower vegetable intake and more problematic mealtime behaviours.	No evidence that child resistance mediated an association between permissive parenting and HbA1c.
López-Morago et al., 2023 [30]	Parental food-choice motives→HbA1c and anthropometric/body-composition outcomes	Bivariate simple linear correlations; unadjusted; *p* ≤ 0.05. Total sample n = 85; analytic n for individual associations: NR. Participants without sociodemographic, clinical, or biochemical data were excluded from the final sample; further missing-data handling: NR.	Familiarity→HbA1c: r = 0.233, *p* < 0.05; maximum reported correlation: r = 0.365 (price→body fat %)	Greater importance placed on food familiarity was weakly associated with higher HbA1c; selected food-choice motives showed weak associations with anthropometric/adiposity measures.	Other FCQ dimensions were not significantly associated with HbA1c or TIR; reported associations were generally weak.
Catamo et al., 2024 [31]	Food neophobia and parental feeding practices→HbA1c categories	Regression analyses adjusted for age, gender, population, and SDS-BMI. Total sample n = 258 (HbA1c < 7.5%: n = 114; 7.5–8.5%: n = 96; >8.5%: n = 48); analytic n for individual regression models: NR. Missing-data handling: NR.	Food neophobia: *p* = 0.04; Pressure to Eat: *p* = 0.0014; Restriction: *p* = 0.0007; Concern about Weight: *p* = 0.010; effect magnitudes/95% CIs NR	Food neophobia and selected parental feeding-practice scores differed across HbA1c-control categories.	Food-liking groups were not significantly associated with glycaemic control.
Lobley et al., 2025 [32]	Feeding practices→HbA1c target status, dietary intake and weight status	Independent-samples *t*-tests and linear models for continuous outcomes; covariate adjustment: NR. Total sample n = 76; HbA1c-target comparison n = 76 (<7.0%: n = 17; ≥7.0%: n = 59); analytic n for individual diet-related associations: NR. Some variables had incomplete data; no imputation or other missing-data handling method was reported.	Covert Restriction: 3.79 ± 0.5 vs. 3.27 ± 0.9, *p* = 0.002; Reward for Eating→vegetables: r = −0.280, *p* = 0.02; →non-core foods: r = 0.320, *p* < 0.01; Reward for Behaviour→non-core foods: r = 0.243, *p* = 0.03; Persuasive Feeding→core foods: r = 0.254, *p* = 0.03	Greater covert restriction was reported among children with HbA1c < 7%; reward-based feeding was associated with lower vegetable and/or greater non-core food intake, whereas persuasive feeding was associated with greater core-food intake.	Other feeding practices were not significantly associated with HbA1c target status; no individual feeding practice was significantly associated with weight status.

BG, blood glucose; BPFAS, Behavioral Pediatrics Feeding Assessment Scale; CGM, continuous glucose monitoring; CHO, carbohydrate; CI, confidence interval; FIML, full information maximum likelihood; HEI-2005, Healthy Eating Index 2005; MICS, Mealtime Interaction Coding System; NR, not reported. Effect estimates are presented as reported in the original studies; 95% CIs were not calculated post hoc when not reported.

**Table 6:** Newcastle–Ottawa Scale for risk of bias assessment of the included reports (scores ≥ 7–9, 4–6, <4 are considered low, intermediate and high risk, respectively).

	Patton, 2006 [22]	Patton, 2009 [23]	Patton (Pump Therapy), 2009 [24]	Patton, 2013 (JAD) [25]	Patton, 2013 [26]	Monaghan, 2015 [27]	Eleanor R. Mackey, 2020 [28]	Kelleher, 2023 [29]	López-Morago, 2023 [30]	Catamo, 2024 [31]	Lobley, 2025 [32]
Study Design	Cross-sectional study	Cross-sectional, Observational, Correlational Study	Cross-sectional Study	Cross-sectional Study	Cross-sectional, Observational Study	Cross-sectional Study	Cross-sectional (baseline data from intervention trial)	Cross-sectional Study	Cross-sectional, Observational Study	Cross-sectional, Observational Study	Cross-sectional Study
**Selection (1)**											
**Selection (2)**											
**Selection (3)**											
**Selection (4)**	**	**	**	**	**	**	**	**	**	**	**
**Comparability (1)**	*				*	*	*	*		*	*
**Outcome (1)**	**	**	**	**	**	**	**	**	**	**	**
**Outcome (2)**	*	*	*	*	*	*	*	*	*	*	*
**Score**	6/9	5/9	5/9	5/9	6/9	6/9	6/9	6/9	5/9	6/9	6/9

* Equals one point and ** equals 2 points, with a total of 9 points. Interpretation: All reports present an intermediate risk of bias. All studies recruited samples from selected clinical centres, which may limit representativeness of the broader population with diabetes (Selection 1). None of the studies included more than 400 participants (Selection 2). None achieved a response rate of 95% or higher (Selection 3). All studies used validated assessment tools (Selection 4). 7/11 studies analysed potential confounding variables (Comparability). In all reports, data collection combined self-administered methods (e.g., questionnaires) with objective record-based data (medical charts, glucose sensors, blood analyses) or independent blinded assessments conducted by trained professionals (Outcome 1): 8/11 combined self-reports with objective records, and 3/11 combined self-reports with independent blinded assessments. In all cases, the statistical methods used were clearly described (Outcome 2).

## Data Availability

No new data were created or analyzed in this study. Data sharing is not applicable to this article.

## Supplementary Materials

The following supporting information can be downloaded at: https://www.mdpi.com/article/10.3390/pediatric18050129/s1, Table S1: PRISMA 2020 checklist.

## Abbreviations

The following abbreviations are used in this manuscript:

ADA	American Diabetes Association
BMI	Body Mass Index
BPFAS	Behavioral Pediatrics Feeding Assessment Scale
CALD	Culturally and Linguistically Diverse
CFQ	Child Feeding Questionnaire
CGM	Continuous Glucose Monitoring
DINE	Dyadic Interaction Nomenclature for Eating
HbA1c	Glycated Haemoglobin
ISPAD	International Society for Pediatric and Adolescent Diabetes
MICS	McMaster Interaction Coding System
NOS	Newcastle–Ottawa Scale
PEOS	Population, Exposure, Outcomes and Study Design
PRISMA	Preferred Reporting Items for Systematic Reviews and Meta-Analyses
PROSPERO	International Prospective Register of Systematic Reviews
T1D	Type 1 Diabetes Mellitus
TIR	Time in range

## Appendix A. Terminology Harmonisation Table

This table harmonises behavioural and conceptual constructs across the eleven studies included in the systematic review to ensure terminological consistency.

**Construct**	**Definition**	**Instruments**	**Studies**
Parental feeding practices	Specific strategies used by parents to influence what, when, and how much a child eats.	CFQ; CFPQ; FPSQ-28	Patton 2009 (pump) [24]; Mackey 2020 [28]; Catamo 2024 [31]; Lobley 2025 [32]
Responsive vs. non-responsive feeding	Feeding behaviours characterised by sensitivity to child hunger/satiety cues versus coercive or instrumental control.	FPSQ-28	Lobley 2025 [32]
Child mealtime behaviours	Observable child behaviours during meals (e.g., refusal, tantrums, distractibility).	BPFAS; DINE; observational coding	Patton 2006–2013 [22,23,24,25,26]; Monaghan 2015 [27]
Parent mealtime behaviours	Parental actions and interaction patterns during meals.	BPFAS; observational coding	Patton 2006–2013 [22,23,24,25,26]
Family mealtime functioning	Emotional climate, structure, task accomplishment and conflict during meals.	MICS	Patton 2009 [23], 2013 [25,26]; Kelleher 2023 [29]
Mealtime structure	Organisation of meal timing, setting, and routines.	FPSQ-28; MICS	Patton 2009 [23], 2013 [25,26]; Lobley 2025 [32]
Food choice motivations	Parental cognitive and motivational drivers influencing food selection.	FCQ	López-Morago 2023 [30]
General parenting style	Broader parenting approach not specific to the feeding context.	Parenting scales	Kelleher 2023 [29]

## Appendix B. Search Strategy

PUBMED

(“Feeding Behaviour”[Mesh] OR “Feeding Behaviour”[tiab] OR “Feeding Behaviours”[tiab] OR “Eating Behaviour”[tiab] OR “Eating Behaviours”[tiab] OR “Feeding-Related Behaviour”[tiab] OR “Feeding Related Behaviour”[tiab] OR “Feeding-Related Behaviours”[tiab] OR “Feeding Patterns”[tiab] OR “Feeding Pattern”[tiab] OR “Feeding Methods”[Mesh] OR “Feeding Methods”[tiab] OR “Feeding Method”[tiab] OR “Feeding styles”[tiab] OR “Feeding practices”[tiab] OR “Feeding attitudes”[tiab] OR “Feeding traits”[tiab] OR “Feeding control”[tiab] OR “parental control”[tiab] OR “Parents”[Mesh] OR “Parents”[tiab] OR “Parent”[tiab] OR “Mothers”[Mesh] OR “Mothers”[tiab] OR “Mother”[tiab] OR “Fathers”[Mesh] OR “Fathers”[tiab] OR “Father”[tiab])

AND

(“Infant”[Mesh] OR “Infant”[tiab] OR “Infants”[tiab] OR “Child”[Mesh] OR “Child”[tiab] OR “Children”[tiab] OR “Adolescent”[Mesh] OR “Adolescent”[tiab] OR “Adolescents”[tiab] OR “Adolescence”[tiab] OR “Teens”[tiab] OR “Teen”[tiab] OR “Teenagers”[tiab] OR “Teenager”[tiab] OR “Youth”[tiab] OR “Youths”[tiab])

AND

(“Diabetes Mellitus, Type 1”[Mesh] OR “Diabetes Mellitus, Type 1”[tiab] OR “DM1”[tiab] OR “Insulin-Dependent Diabetes Mellitus”[tiab] OR “Type 1 Diabetes Mellitus”[tiab] OR “Insulin-Dependent Diabetes Mellitus 1”[tiab] OR “Insulin Dependent Diabetes Mellitus 1”[tiab] OR “Type 1 Diabetes”[tiab])

WOS

(TS = (“Diabetes Mellitus Type 1” OR “DM1” OR “Type 1 Diabetes Mellitus” OR “Insulin Dependent Diabetes Mellitus” OR “Type 1 Diabetes” OR “IDDM”))

AND

(TS = (Infant OR Child OR Children OR Adolescent OR Adolescence OR Teen OR Teenager OR Youth))

AND

(TS = (“Feeding Behaviour” OR “Eating Behaviour” OR “Feeding Related Behaviour” OR “Feeding Pattern” OR “Feeding Method” OR “Feeding styles” OR “Feeding practices” OR “Feeding attitudes” OR “Feeding traits” OR “Feeding control” OR “parental control”))

SCOPUS

((TITLE-ABS-KEY (“dm1”)) OR (TITLE-ABS-KEY (“diabetes mellitus type 1”)) OR (TITLE-ABS-KEY (insulin AND dependent AND diabetes AND mellitus)) OR (TITLE-ABS-KEY (“type 1 diabetes”)) OR (TITLE-ABS-KEY (iddm)))

AND

((TITLE-ABS-KEY (infant)) OR (TITLE-ABS-KEY (child)) OR (TITLE-ABS-KEY (children)) OR (TITLE-ABS-KEY (adolescent)) OR (TITLE-ABS-KEY (adolescence)) OR (TITLE-ABS-KEY (teen)) OR (TITLE-ABS-KEY (youth)) OR (TITLE-ABS-KEY (teenager)))

AND

((TITLE-ABS-KEY (“parental control”)) OR (TITLE-ABS-KEY (“Feeding control”)) OR (TITLE-ABS-KEY (“Feeding traits”)) OR (TITLE-ABS-KEY (“Feeding attitudes”)) OR (TITLE-ABS-KEY (“Feeding practices”)) OR (TITLE-ABS-KEY (“Feeding styles”)) OR (TITLE-ABS-KEY (“Feeding Method”)) OR (TITLE-ABS-KEY (“Feeding Pattern”)) OR (TITLE-ABS-KEY (“Feeding Related Behaviour”)) OR (TITLE-ABS-KEY (“Eating Behaviour”)) OR (TITLE-ABS-KEY (“Feeding Behaviour”)))
